# Psychometric properties of the Chinese version of Five Facet Mindfulness Questionnaire—short form in cancer patients: a Bayesian structural equation modeling approach

**DOI:** 10.1186/s12955-021-01692-1

**Published:** 2021-02-10

**Authors:** Ted C. T. Fong, Adrian H. Y. Wan, Venus P. Y. Wong, Rainbow T. H. Ho

**Affiliations:** 1 Centre on Behavioral Health, University of Hong Kong, 2/F, The HKJC Building for Interdisciplinary Research, 5 Sassoon Road, Pokfulam, Hong Kong; 2 Department of Social Work and Social Administration, The University of Hong Kong, Pokfulam, Hong Kong

**Keywords:** Assessment, Awareness, Factor analysis, Mindfulness, Validation

## Abstract

**Background:**

Mindfulness has emerged as an important correlate of well-being in various clinical populations. The present study evaluated the psychometric properties of the 20-item short form of the Five Facet Mindfulness Questionnaire (FFMQ-SF) in the Chinese context.

**Methods:**

The study sample was 127 Chinese colorectal cancer patients who completed the FFMQ-SF and validated physical and mental health measures. Factorial validity of the FFMQ-SF was assessed using Bayesian structural equation modeling (BSEM) via informative priors on cross-loadings and residual covariances. Linear regression analysis examined its convergent validity with the health measures on imputed datasets.

**Results:**

The five-factor BSEM model with approximate zero cross-loadings and one residual covariance provided an adequate model fit (PPP = 0.07, RMSEA = 0.06, CFI = 0.95). Satisfactory reliability (ω = 0.77–0.85) was found in four of the five facets (except nonjudging). Acting with awareness predicted lower levels of perceived stress, negative affect, anxiety, depression, and illness symptoms (β = − 0.37 to − 0.42) and better quality of life (β = 0.29–0.32). Observing, nonjudging, and nonreacting did not show any significant associations (*p* > .05) with health measures. Acting with awareness was not significantly correlated (*r* < 0.15) with the other four facets.

**Conclusion:**

The present findings provide partial support for the psychometric properties of the FFMQ-SF in colorectal cancer patients. The nonjudging facet showed questionable validity and reliability in the present sample. Further studies with larger sample sizes are needed to elucidate the viability of FFMQ-SF as a measure of mindfulness facets in cancer patients.

## Background

Colorectal cancer is the third most frequent type of cancer around the world [1]. In Hong Kong, colorectal cancer ranked the second most common in accounting for over one-sixth of the new cancer cases in 2016 [2]. Patients diagnosed with colorectal cancer typically undergo surgical treatments (radical resection) and adjuvant chemotherapy and radiotherapy. Colorectal cancer patients are at risk of both physical symptoms (fatigue, lethargy, and insomnia) and emotional symptoms (loss of control, anxiety, depression, and fear of recurrence) following cancer treatments [3].

Mindfulness-based interventions have received tremendous interest from researchers and practitioners in health settings [4]. Mindfulness is a psychological construct that originated from the West. It involves bringing one’s complete attention to experiences that occur in the present moment in a non-judgmental and accepting way [5]. Evidence from a meta-analysis [6] has found beneficial effects of mindfulness on physical and mental well-being in clinical populations. Among cancer patients, mindfulness interventions have shown improvements in psychosocial adjustment [7], cognitive functioning [8], cortisol slopes, and telomerase length [9]. A viable and valid tool for measuring mindfulness is essential for evaluating the mechanisms of the mindfulness effects [10].

A review [11] found eight available self-report measures of mindfulness, with examples such as the Kentucky Inventory of Mindfulness Skills [12], Mindful Attention Awareness Scale [13], Freiburg Mindfulness Inventory [14], Cognitive and Affective Mindfulness Scale-Revised [15], and Southampton Mindfulness Questionnaire [16]. The Five Facet Mindfulness Questionnaire (FFMQ) [17] was a 39-item multifaceted scale developed from the above five validated mindfulness questionnaires. The FFMQ has a comprehensive conceptualization by integrating the five validated mindfulness scales via measuring five distinct mindfulness facets. The FFMQ has shown good psychometric properties in terms of construct validity and reliability in a systematic review of mindfulness instruments [18]. A 20-item short form of the FFMQ (FFMQ-SF) was developed under comprehensive criteria in a community sample of healthy adults and a clinical sample with psychological distress [19].

Both the FFMQ and FFMQ-SF have been validated primarily in samples of healthy adults, meditators, and depressive patients [20–26]. In the context of cancer patients, the only psychometric study [27] revealed a six-factor structure rather than the original five-factor structure for the FFMQ in a sample of prostate cancer patients. To our knowledge, no existing studies have examined the validity and reliability of the FFMQ-SF in cancer populations. The FFMQ has shown measurement non-invariance in the form of scalar non-invariance (different intercepts) between clinical and non-clinical samples [28] and metric non-invariance (different loadings) between meditators and non-meditators [29]. These findings imply that the factor structure and measurement parameters of the FFMQ-SF could differ across the clinical contexts. There is a practical need to evaluate the psychometric properties of the FFMQ-SF to authenticate its use in the cancer populations.

The majority of validation studies on the FFMQ-SF focused on confirmatory factor analysis via the maximum likelihood approach. This traditional approach depends on the unrealistic assumptions of zero cross-loadings [30] and limits the researchers’ ability to investigate cross-loadings and residual covariance parameters [31]. This approach could lead to inflated model rejections and contribute to the discrepancy in the dimensionality of the FFMQ in the previous psychometric study in cancer samples [27]. Bayesian structural equation modeling (BSEM) is a new approach that can flexibly specify these minor parameters to be approximately zero using informative priors [32]. The present study aimed to evaluate the psychometric properties of the FFMQ-SF in a sample of colorectal cancer patients. The present study hypothesized that the five-factor structure would provide adequate factorial validity in terms of an adequate model fit, satisfactory reliability, and convergent validity with health outcomes.

## Methods

### Participants and procedures

The study sample was a total of 127 Chinese patients with colorectal cancer. The participants were recruited by convenience sampling via doctors’ referrals and newsletter advertisements in cancer patient resource centers in two local hospitals and three community organizations servicing cancer patients in Hong Kong. Trained research assistants conducted the screening based on the following inclusion criteria: diagnosis of stage 0 to III colorectal cancer, expected survival time of at least 12 months, Chinese speaking, aged 18 or above, and 0.5 to 5 years following cancer treatment. The exclusion criteria were the presence of severe cachexia, bone pain, nausea, or diagnosis of a major psychiatric disorder or other forms of cancer. All of the participants provided voluntary written informed consent before joining the study. The participants completed a set of paper-and-pencil questionnaires comprising the FFMQ-SF and validated self-report measures of perceived stress, affect, anxiety, depression, illness symptoms, and quality of life. Table 1 summarizes the psychometric information (number of items, number of factors, score range, and reliability) of the adopted scales. Completion of the questionnaires took around 20–25 min. Ethical approval was obtained from the human research ethics committee of the author’s university.

**Table 1:** Summary of psychometric information for the measurement scales in this study

Measurement scale	N	Dimension	Likert format	Score range	Α
Five Facet Mindfulness Questionnaire	20	5-factor	1–5	Average (1–5)	.69–.85
Perceived Stress Scale	10	1-factor	0–4	Sum (0–40)	.76
Positive and Negative Affect Schedule	20	2-factor	1–5	Sum (10–50)	.89
Hospital Anxiety and Depression Scale	14	2-factor	0–3	Sum (0–21)	.78–.87
Memorial Symptom Assessment Scale	32	1-factor	0–4	Average (0–4)	.94
Short-form 12 Health Survey	12	2-factor	various	Sum (0–100)	.72–.77

N = Total number of items; α = Cronbach’s alpha

### Measurements

#### Mindfulness

The Chinese version of the FFMQ-SF is a 20-item self-report questionnaire [19] that assesses five facets of mindfulness: observing one’s reaction (observing), ability to describe this reaction (describing), acting with awareness, nonjudging of inner experience (nonjudging), and nonreacting to inner experience (nonreacting). Four items measure each facet. The 20 items were selected from the original 39-item FFMQ using criteria such as maintenance of reliability, item-total correlation of at least 0.40, and minimum cross-loadings. Participants rate the degree to which each statement applies to them on a 5-point Likert scale (1 = never, 5 = very often). Items on two facets (acting with awareness and nonjudging) are reversely coded. Each facet score is the average of the four items, with higher scores (theoretical range = 1–5) indicating higher mindfulness levels. The original 5-factor structure of the FFMQ-SF can be found in the Additional file 1 as reference.

#### Perceived stress

The Chinese version [33] of the Perceived Stress Scale [34] measures the participants’ stress level over the past week. This 10-item scale asks about the degree to which the respondent appraises life events as stressful on a 5-point (0 = never, 4 = very often) Likert scale. Higher scores (theoretical range = 0–40) indicate higher levels of perceived stress. The scale showed a satisfactory level of reliability (*α* = 0.76) in the present study.

#### Affect

The Chinese version [35] of the 20-item Positive and Negative Affect Schedule [36] was used to assess the participants’ ambient moods over the past two weeks. The scale consists of 10 items each to measure positive affect (e.g., active, determined, inspired, interested) and negative affect (e.g., guilty, irritable, nervous, upset) on a 5-point (1 = never, 5 = very often) Likert scale. The higher the score (theoretical range = 10–50) on a subscale, the more the respondent experienced that affective state. Both positive and negative affect showed good reliability (*α* = 0.89) in the present study.

#### Anxiety and depression

The Chinese version [37] of the Hospital Anxiety and Depression Scale [38] was used to measure anxiety and depressive symptoms over the past week. This 14-item scale consists of two subscales: anxiety (seven items) and depression (seven items) measured on a 4-point (0–3) Likert scale. The item sum scores provide the total subscale scores and higher scores (theoretical range = 0–21) indicate higher distress. The scale showed good reliability (*α* = 0.78–0.87) in this study.

#### Cancer-related illness symptoms

The Chinese version [39] of the Memorial Symptom Assessment Scale [40] was used to measure participants’ cancer-related symptom distress. This instrument evaluates 32 symptoms (such as pain, nausea, insomnia, diarrhea, and drowsiness) experienced over the past week regarding frequency, severity, and distress, each measured on a 4-point Likert scale. In case of absence of experience of a symptom, each domain’s score and the symptom overall is scored as zero. If a symptom is experienced, the score is calculated as the average of the frequency, severity, and distress domains. The symptom scores for all 32 symptoms are averaged to produce the total scale score (theoretical range = 0–4). The total score showed excellent reliability (*α* = 0.94) in the present study.

#### Quality of life (QoL)

The Chinese version [41] of the short-form Health Survey (SF-12) is a commonly used self-report measure of health-related QoL [42]. The 12 items produce summary scores for the physical and mental health domains using standardized scoring (theoretical range = 0–100). The scale showed acceptable levels of reliability (*α* = 0.72–0.77) in the present study. A previous study in Hong Kong [43] established separate population norms for 1493 persons without chronic diseases (mean physical QoL = 53.2, *SD* = 6.3; mean mental QoL = 50.6, *SD* = 8.8) and 917 persons with chronic diseases (mean physical QoL = 44.7, *SD* = 11.0; mean mental QoL = 49.1, *SD* = 10.5). The respondents’ physical and mental QoL were compared to these population norms to understand their well-being levels.

### Statistical analysis

Exploratory factor analysis (EFA) was first conducted to determine the dimensionality of the FFMQ-SF by comparing models with an increasing number of factors (from 2 to 6). EFA is considered a less-restrictive and more realistic variant of confirmatory factor analysis by allowing cross-loadings. The underlying factor structure was evaluated via BSEM [30] using Mplus 8.4. The BSEM approach does not assume normal distributions for the model parameters [32], but instead allows direct estimation of the posterior distributions based on the data and prior distribution. The Bayesian approach was found to outperform the maximum likelihood approach in terms of accuracy of parameter estimates in factor analysis with small sample sizes (N < 200) [44]. In the present study, all of the 20 items showed skewness and kurtosis values less than one and fulfilled the normality testing requirements for Bayesian estimation. The primary factor loadings should be at least 0.40 and substantial factor loadings should have magnitudes of ≥ 0.50.

The rationale and methodological details of BSEM are available to the readers in relevant literature [30–32, 45]. In the present study, we fitted the BSEM models following a series of prior specifications: 1) uninformative priors, 2) informative priors for approximate zero cross-loadings, and 3) informative priors for approximate zero residual covariances. Sensitivity analysis was conducted by varying the informative priors in the BSEM analysis [31]. The prior variance for the cross-loadings varied from 0.01 to 0.04, and the degrees of freedom (d) ranged from 10 to 30. Model estimation was conducted using Markov chain Monte Carlo methods with two chains and at least 20,000 iterations. The first half of the iterations were used as burn-in phase and discarded, and the latter half derived the empirical distribution of the model parameters. Model convergence was checked via the potential scale reduction criterion [46] with values of < 1.05 implying small between-chain variation.

The model fit of the BSEM models was assessed using posterior predictive checking via the posterior predictive *p* value (PPP), root mean square error of approximation (RMSEA), and comparative fit index (CFI). A positive lower 95% posterior predictive (PP) limit denoted a poor model fit between the real and replicated data. For the PPP, a small value (< 0.05) denotes rejection of an exact model fit with a higher value indicating a better fit. The prior posterior predictive *p* value (pppp) was used to evaluate the tenability of the minor parameters with small values (pppp < 0.05) against the prior specification. Comparison of the BSEM models was based on the deviance information criterion (DIC), which penalizes model deviance with the estimated number of model parameters and avoids model overfitting. A lower DIC value denotes a better model fit with greater parsimony. The optimal BSEM model should provide a good model fit (PPP > 0.05, RMSEA ≤ 0.06, and CFI ≥ 0.95) and have the lowest DIC. Traditional factor analysis was conducted under the maximum likelihood approach to supplement the Bayesian results. The results under the maximum likelihood approach are shown in Additional file 2 and Additional file 3 displays the CFA model of the 5-factor structure.

The present study examined the convergent validity of the FFMQ-SF under a two-step approach. The first step used multiple imputations to generate 50 imputed datasets for the plausible values of FFMQ-SF factors in the BSEM model. An interval of 100 iterations was used for thinning to derive the imputed datasets. The second step performed univariate regression analysis to select potential demographic factors of the derived FFMQ-SF factor scores at *p* < 0.10 level. The univariate regression model included gender, age, cancer stage, education level, marital status, comorbid illness, and income as potential model covariates. The third step conducted multivariate regression analysis by regressing the physical and mental health measures (perceived stress, affect, anxiety, depression, illness symptoms, and quality of life) on the FFMQ-SF factor scores using the imputed datasets. The regression analyses were averaged over the 50 imputed datasets to improve the results’ reliability.

The composite reliability of the FFMQ-SF factors was assessed by the Omega (ω) coefficient, with values of ω ≥ 0.75 indicative of acceptable reliability [47]. The participants completed the FFMQ-SF again four weeks later to evaluate the test–retest reliability. Intraclass correlation coefficient (ICC) values above 0.75 indicated good test–retest reliability [48]. The amount of missing data was minimal in the FFMQ-SF, with 125 of the 127 participants completing all 20 items. Eight participants had missing data on some of the health measures and around one-tenth of the sample did not provide information on their cancer stage or income (N = 12–16). Missingness in cancer stage and income was significantly and positively associated (*r* = 0.20, *p* = 0.023). Participants who reported missing data in cancer stage or income were likely to be older, have lower education level, have lower levels of perceived stress and negative affect, and have higher mental quality of life. Missing data were handled using full information maximum likelihood under the missing-at-random assumption [49].

## Results

### Sample profile

The majority of the sample was female (58%), married (76%), and had at least an upper secondary education (64%). The sample mean age was 63.8 years (SD = 8.9) and around two-third had been diagnosed with stage II or III colorectal cancer. Most participants (96%) received surgery treatment and less than half received chemotherapy or radiotherapy treatment. The median monthly income of the sample was 5.5 thousand HKD. Around one-fourth of the sample reported comorbid illness such as hypertension and diabetes. Table 2 lists the sample means (SD) for perceived stress, positive affect, negative affect, anxiety, depression, illness symptoms, physical QoL, and mental QoL. The present sample showed significantly lower levels of physical QoL than persons without chronic diseases (*t* = − 13.7, *p* < 0.01, *d* = 1.56) and persons with chronic diseases (*t* = − 1.99, *p* < 0.05, *d* = 0.15). The present sample displayed comparable levels (*p* > 0.05, *d* < 0.15) of mental QoL as persons with and without chronic diseases.

**Table 2:** Demographic and clinical profiles of the patients

Variable	N (%)
Female/male	74 (58)/53 (42)
*Education level*	
≤ 9 years/ ≥ 10 years	45 (36)/82 (64)
*Marital status*	
Single/married	31 (24)/96 (76)
*Cancer stage*	
I/II/III	35 (32)/33 (30)/43 (38)
Having comorbid illness	33 (26)
*Chemotherapy or radiotherapy*	
Yes/no	56 (44)/70 (56)

	Mean (SD)
Age (years)	63.8 (8.9)
Average monthly income (k)	15.0 (32.6)
Perceived stress	17.7 (4.5)
Positive affect	28.5 (6.7)
Negative affect	19.2 (6.2)
Anxiety	5.5 (4.0)
Depression	5.3 (3.7)
Illness symptoms	0.59 (0.47)
Physical quality of life	43.1 (8.1)
Mental quality of life	50.6 (8.0)

*SD* standard deviation

### Dimensionality

For the Bayesian EFA models (Table 3), the two-, three-, and four-factor models provided mediocre fits to the data with positive lower PP limits and PPP < 0.001. The five-factor EFA model showed an adequate model fit with a negative lower PP limit and lower DIC. The six-factor EFA model provided the best model fit in terms of lower PP limit and DIC. However, the sixth factor had only one significant factor loading and was not correlated with the other five factors, suggesting the potential over-extraction of factors. Overall, the results lent support to the presence of five factors for the FFMQ-SF.

**Table 3:** Fit indices of the 2-factor to 6-factor EFA models for the FFMQ-SF

EFA model	#	2.5% PPL	97.5% PPL	PPP	DIC
2-factor	79	222.6	327.9	.000	6668
3-factor	97	133.3	243.1	.000	6598
4-factor	114	44.7	157.8	.000	6526
5-factor	130	− 0.3	118.4	.027	6500
6-factor	145	− 23.8	94.6	.115	6488

N = 127; # = number of free parameters

*PPL* posterior predictive limit, *PPP* posterior predictive *p* value, *DIC* deviance information criterion

### Factorial validity

Table 4 shows the fit indices of the five-factor BSEM models with various prior specifications. Using uninformative priors, Model 1 provided a poor fit (high lower PP limit and PPP < 0.001). Informative priors were added for approximate zero cross-loadings in Models 2–4. Model 2 was rejected by the positive lower PP limit (PPP < 0.01). Model 3 showed a good prior PP *p* value (> 0.5) and a negative lower PP limit. Increasing the prior variance to 0.04 in Model 4 resulted in trivial improvements in the PP limits and PPP, but not the RMSEA or CFI. Because Model 3 had a lower DIC and allowed smaller cross-loadings (± 0.28 instead of ± 0.40) than Model 4, the prior variance for the cross-loadings was set to 0.02 in the subsequent analysis. In Model 3, item 5 (“shouldn’t be feeling the way I’m feeling”) did not load significantly on “nonjudging” (λ = 0.23, 95% CI = − 0.05 to 0.50) but instead loaded on “act with awareness” (λ = 0.30, 95% CI = 0.13 to 0.46). After this re-specification, Model 5 showed a slightly better model fit than Model 3 in terms of the PP limits, PPP, and DIC. These models failed to provide an adequate model fit with PPP < 0.05, RMSEA > 0.06, and CFI < 0.95, which necessitated further investigation of the model misfit source.

**Table 4:** Fit indices of the 5-factor BSEM models with different priors for the FFMQ-SF

Model	Prior specification	#	pD	pppp	2.5% PPL	97.5% PPL	PPP	DIC	RMSEA	CFI
1	Uninformative	70	72	–	100.4	204.3	.000	6403	0.091	0.84
*Informative priors on cross-loadings*										
2	var = 0.01	150	100	.12	12.5	132.7	.008	6352	0.072	0.92
3	var = 0.02	150	108	.61	− 3.0	117.5	.030	6343	0.066	0.93
4	var = 0.04	150	115	.94	− 8.0	115.7	.044	6347	0.066	0.94
5	Revised Model 3	150	107	.70	− 4.8	111.7	.037	6339	0.065	0.94
*Informative priors on residual covariance*										
6	d = 10	340	211	.44	− 75.5	50.9	.662	6377	0.010	1.00
7	d = 20	340	209	.20	− 78.9	42.2	.726	6369	0.000	1.00
8	d = 30	340	214	.06	− 73.6	50.0	.644	6381	0.020	0.99
*Informative priors on cross-loadings with one residual covariance*										
9	Revised Model 5	151	108	.75	− 14.9	97.6	.073	6329	0.060	0.95

N = 127; # = number of free parameters; pD = estimated number of parameters; pppp = prior posterior predictive *p* value; PPL = posterior predictive limit; PPP = posterior predictive *p* value; DIC = Deviance information criterion; RMSEA = Root mean square error of approximation; CFI = comparative fit index; d = degree of freedom

Informative priors were added on the items’ residual covariances. Models 6–8 provided an exact fit to the data with negative lower PP limits and PPP ~ 0.5. However, these models showed higher DICs than Model 5, with a great increase in the number of estimated parameters. Among the 190 specified residual correlations, only three were statistically significant: item 6 (“step back”) with item 17 (“visual elements”) (*r* = 0.28, 95% CI = 0.03 to 0.51); item 8 (“make judgments”) with item 18 (“experiences”) (*r* = 0.29, 95% CI = 0.02 to 0.51); and item 6 (“step back”) with item 19 (“notice and let go”) (*r* = − 0.33, 95% CI = − 0.56 to − 0.09). Models 6–8 showed a consistent pattern of results regarding the three significant residual correlations.

Out of these three residual correlations, only the last residual correlation contained items with a shared underlying factor (Nonreacting). The re-specification adopted only this residual correlation but not the other two. Model 9 with this added residual correlation between item 6 and item 19 showed an adequate model fit with a negative lower PP limit, PPP > 0.05, RMSEA ≤ 0.06 and CFI ≥ 0.95, and the lowest DIC out of the nine models. As shown in Table 5, 15 out of the 20 items showed statistically significant and substantial (λ = 0.54–0.96, *p* < 0.05) factor loadings. The main factor loadings for the remaining five items (Item 5, 8, 15, 16, and 19) were statistically significant but less than 0.50 (λ = 0.38–0.49, *p* < 0.05). All of the cross-loadings were small and fell within the specified range of ± 0.28.

**Table 5:** Factor loadings (with 95% CI) of revised BSEM model for the FFMQ-SF in Model 9

Item no	Observing	Describing	Act with awareness	Nonjudging	Nonreacting
7. Sensations	.54* (.34–.74)	.05 (− .14 to .22)	− .03 (− .19 to .14)	.01 (− .18 to .18)	.12 (− .07 to .31)
10. Sound	.88* (.69 to 1.09)	− .10 (− .30 to .09)	− .03 (− .22 to .16)	.07 (− .13 to .28)	− .09 (− .30 to .11)
14. Smell and aroma	.83* (.65 to 1.02)	.02 (− .17 to .21)	.01 (− .17 to .20)	.03 (− .17 to .22)	− .10 (− .30 to .09)
17. Visual elements	.60* (.40 to .80)	.06 (− .13 to .24)	.03 (− .14 to .19)	− .06 (− .25 to .13)	.14 (− .06 to .33)
1. Describe feelings	− .12 (− .31 to .08)	.96* (.78 to 1.17)	− .03 (− .23 to .16)	− .03 (− .23 to .17)	− .06 (− .26 to .14)
3. Beliefs and opinions	− .01 (− .19 to .17)	.85* (.68 to 1.03)	.01 (− .17 to .18)	.01 (− .18 to .19)	− .01 (− .20 to .18)
15. Terribly upset	.09 (− .08 to .26)	.41* (.21 to .61)	.13 (− .02 to .29)	.13 (− .05 to .30)	.18 (− .01 to .37)
18. Experiences	.09 (− .09 to .27)	.70* (.50 to .89)	− .03 (− .19 to .14)	− .01 (− .18 to .18)	− .02 (− .22 to .17)
2. Mind wanders off	− .06 (− .24 to .12)	.07 (− .11 to .25)	.81* (.69 to .93)	.12 (− .06 to .30)	− .03 (− .22 to .16)
4. Daydreaming	.05 (− .13 to .23)	− .02 (− .21 to .16)	.79* (.66 to .91)	.03 (− .15 to .21)	− .03 (− .22 to .17)
11. Easily distracted	.12 (− .07 to .31)	.06 (− .13 to .24)	.75* (.61 to .89)	− .22* (− .40 to − .03)	.05 (− .14 to .25)
20. Not paying attention	− .08 (− .26 to .10)	− .04 (− .22 to .15)	.76* (.63 to .89)	.09 (− .09 to .27)	− .03 (− .23 to .16)
5. Shouldn’t be feeling	− .07 (− .25 to .11)	− .10 (− .28 to .08)	.47* (.28 to .63)	− .11 (− .29 to .07)	− .01 (− .19 to .19)
8. Make judgments	.12 (− .06 to .30)	.20* (.01 to .37)	.04 (− .13 to .20)	.38* (.16 to .63)	.08 (− .13 to .28)
13. Shouldn’t be thinking	.01 (− .22 to .23)	− .10 (− .32 to .15)	− .01 (− .23 to .21)	.93* (.65 to 1.16)	.05 (− .20 to .30)
16. Bad emotions	.01 (− .18 to .20)	.12 (− .07 to .31)	− .07 (− .24 to .10)	.48* (.24 to .75)	.01 (− .21 to .22)
6. Step back	− .01 (− .20 to .19)	− .05 (− .25 to .13)	− .04 (− .23 to .14)	.01 (− .20 to .21)	.70* (.45 to .95)
9. Pause immediately	− .10 (− .29 to .10)	.04 (− .16 to .23)	− .05 (− .24 to .13)	− .01 (− .22 to .19)	.79* (.55 to 1.04)
12. Feel calm soon	.01 (− .18 to .19)	− .02 (− .21 to .16)	.06 (− .12 to .22)	.12 (− .10 to .31)	.66* (.42 to .90)
19. Notice and let go	.12 (− .07 to .31)	.06 (− .14 to .25)	.01 (− .16 to .18)	.01 (− .21 to .20)	.49* (.21 to .77)

N = 127; Bolded loadings were freely estimated with diffuse priors and all remaining cross-loadings were estimated with informative priors N (0, 0.02)

*p < 0.05

### Reliability and factor correlations

As shown in Table 6, four of the five facets (except nonjudging) displayed satisfactory omega coefficients (ω = 0.77–0.85). Good test–retest reliability (ICC = 0.75–0.87) was found for the FFMQ-SF factors across the 4-week interval. From the BSEM results, four of the five facets showed positive and moderate correlations (*r* = 0.30–0.57, *p* < 0.05). Acting with awareness was not associated with the other factors (*r* = 0.00–0.15, *p* > 0.05). The factor correlations derived in EFA via the robust maximum likelihood estimator displayed a similar pattern.

**Table 6:** Descriptive statistics, reliability, and intercorrelations of the FFMQ-SF factors

Factor	Mean	SD	ω	ICC (95% CI)	Factor correlations				
					1	2	3	4	5
1. Observing	3.01	1.05	.82	.83 (.75–.88)		.42*	.02	.21*	.36*
2. Describing	2.96	1.00	.85	.80 (.71–.86)	.51*		.14	.14	.49*
3. Acting with awareness	3.61	0.82	.85	.87 (.81–.91)	.00	.15		.05	.11
4. Nonjudging	3.02	0.94	.68	.75 (.63–.83)	.37*	.30*	.02		.40*
5. Nonreacting	2.96	0.83	.77	.78 (.68–.85)	.46*	.51*	.12	.57*	

N = 127; SD = standard deviation; ω = omega coefficient; ICC = intraclass correlation coefficient for absolute agreement; CI = confidence interval. Factor correlations above and below the diagonal were derived via robust maximum likelihood and Bayesian estimators, respectively

*p < .05

### Convergent validity

The upper portion of Table 7 shows the standardized estimates of the FFMQ-SF factors on the potential demographic factors in the univariate regression analysis. Age, gender, marital status, and comorbid illness did not have any significant effects (*p* > 0.05) on the FFMQ-SF factors. Participants at an advanced cancer stage scored higher on acting with awareness and those with higher incomes scored lower on observing and describing. More educated participants displayed higher levels of describing and nonreacting.

**Table 7:** Results of univariate and multivariate regression analyses among the FFMQ-SF factors with potential demographic factors and health outcomes

Univariate	Observing	Describing	Awareness	Nonjudge	Nonreact	
Covariates	β (SE)	β (SE)	β (SE)	β (SE)	β (SE)	
Gender	.07 (.10)	.07 (.10)	− .08 (.10)	.03 (.10)	.02 (.10)	
Age	− .09 (.09)	− .16 (.10)	.12 (.10)	.12 (.11)	.11 (.09)	
Cancer stage	− .09 (.10)	.04 (.11)	.18* (.09)	− .09 (.11)	.06 (.11)	
Education	.15 (.11)	.24* (.10)	.10 (.11)	.17 (.11)	.27* (.10)	
Marital status	− .03 (.10)	.05 (.10)	.03 (.11)	− .10 (.10)	− .02 (.11)	
Comorbid illness	.10 (.10)	.09 (.10)	.01 (.10)	− .03 (.10)	.10 (.10)	
Income	− .20* (.08)	− .23* (.09)	− .01 (.11)	− .05 (.12)	− .17 (.09)	

Multivariate	Observing	Describing	Awareness	Nonjudge	Nonreact	
Health outcomes	β (SE)	β (SE)	β (SE)	β (SE)	β (SE)	ΔR^2^
Perceived stress	.00 (.12)	.05 (.14)	− .42* (.08)	.01 (.14)	− .16 (.15)	19.3%
Positive affect	− .06 (.15)	.38* (.13)	.13 (.11)	.25 (.13)	− .02 (.15)	26.3%
Negative affect	− .07 (.13)	.06 (.14)	− .40* (.09)	.01 (.13)	− .11 (.14)	18.7%
Anxiety	.09 (.14)	.08 (.14)	− .40* (.10)	.06 (.13)	− .26 (.15)	20.7%
Depression	.11 (.14)	− .15 (.12)	− .38* (.09)	− .03 (.13)	− .06 (.15)	21.1%
Illness symptoms	− .06 (.13)	.19 (.12)	− .40* (.08)	.09 (.12)	− .17 (.14)	18.0%
Physical QoL	.05 (.15)	− .08 (.14)	.25* (.09)	.03 (.13)	− .07 (.16)	6.5%
Mental QoL	.03 (.13)	.04 (.13)	.32* (.09)	.16 (.13)	− .01 (.15)	14.2%

N = 127; β = standardized regression estimates; SE = standard error; QoL = quality of life; ΔR^2^ = incremental R^2^ of the health outcomes explained by the FFMQ factors

*p < .05

The lower portion of Table 7 shows the standardized estimates of the physical and mental health outcomes on the FFMQ-SF factors. Observing, nonjudging, and nonreacting did not significantly predict any of the health outcomes (*p* > 0.05). Describing significantly predicted greater positive affect (β = 0.38, *p* < 0.05). Acting with awareness was a significant predictor of lower perceived stress, negative affect, anxiety, depression, and illness symptoms (β = − 0.38 to − 0.42, *p* < 0.05), and better quality of life (β = 0.25 to 0.32, *p* < 0.05). As shown in Table 7, the FFMQ facets explained an additional 6.5% to 18.0% of the variance in the physical health outcomes and 14.2% to 26.3% of the variance in the mental health outcomes.

## Discussion

The present study examined the psychometric properties of the FFMQ-SF in a Chinese sample of colorectal cancer patients using the Bayesian approach. The 5-factor structure supported by EFA was rejected by CFA under the maximum likelihood approach in the same sample. The rejection could be attributed to the stringent assumption of exact-zero cross-loadings in the CFA model, which failed to properly account for the small cross-loadings that exist in the EFA model. The Bayesian results supported the five-factor model with approximate zero cross-loadings to represent the underlying structure. Only two of the 80 cross-loadings were statistically significant, and all of them were smaller than 0.28 (M + 2 SD). However, five out of the 20 items did not display substantial loadings (λ < 0.50) on the respective factor. In particular, two of the three items (items 8 and 16) in the nonjudging facet did not have substantial loadings. These factor loadings likely contributed to the lower composite reliability (ω = 0.68) for this factor. These results raise concerns over the construct validity of this factor and its use in the present sample. Though specification of informative priors for residual covariances led to an exact fit, the significantly increased number of estimated parameters resulted in a less parsimonious model compared to Model 9.

Interestingly, item 5 (“I tell myself I should not be feeling the way I am feeling”) belonged to the acting with awareness facet instead of the nonjudging facet. This discrepancy could be attributed to the translation issue of the FFMQ-SF. The phrase “the way” might not be translated into Chinese accurately. Despite sharing similar wordings with item 13 (“should not be thinking the way I am thinking”), the two items loaded on separate factors. The word “should” in these items implies the awareness of experiencing a specific thought or feeling. Interpretation of the experience is more important than the experience itself and results in corresponding feelings [50]. Awareness of habitual cognitive patterns leading to different experiences is considered an adjustable mental capacity trainable through mindfulness practice. Awareness of thought might be deemed more manageable with alertness toward the habitual thinking pattern. In comparison, feelings tend to arise spontaneously as an outcome that might be considered a recognizable rather than a controllable condition. Thus, item 5 could manifest as recognizing the mindless moments while item 13 could represent a nonjudging cognitive capacity.

Acting with awareness consistently predicted various health measures and their close linkages are in line with previous findings [19, 23, 27]. There may be differences in the mental activities assessed by the FFMQ-SF facets. Acting with awareness measures the mindless, distracted moments in daily life through reverse-scored items. People without exposure to or understanding mindfulness may understand such awareness as irritations in daily life [51]. Novice practitioners are commonly surprised by the mindless moments that occur with a heightened sense of awareness. The other four facets appear to measure the capacity to be trained through exposure to mindfulness practices. Because the participants in this study did not have such exposure, they might have found the other four facets less comprehensible than acting with awareness. Differential semantic understanding and interpretations of the items may lead to response biases across mindfulness experience [52].

### Practical implications

Researchers [11, 53] have expressed doubts about the construct validity of the FFMQ and whether it could measure all relevant aspects of mindfulness. Grossman [51] questioned whether the describing facet, which assesses the ability to describe an experience verbally, is an essential mindfulness dimension. The lack of significant associations between acting with awareness and the other four facets in the present sample resembles previous findings [19]. The observing facet of the FFMQ-SF focuses on bodily sensation and external perception, and only emotional awareness was found to correlate with psychological symptoms [54]. The lack of item coverage of the observing facet on emotional awareness could explain the lack of association between this facet and acting with awareness. Similarly, the nonjudging facet did not show any significant associations with the covariates or health outcomes in the multivariate regression. The failure to demonstrate adequate convergent validity for this facet could partly be attributed to its low composite reliability.

In the previous psychometric study on FFMQ in prostate cancer patients [27], acting with awareness emerged as two separate and moderately correlated factors of awareness and attention. On the one hand, the sixth factor could reflect differences in mindfulness’s conceptualization in the cancer population. On the other hand, the present study did not reveal such findings in the FFMQ-SF. The discrepancy could be attributed to differences in the cancer type (prostate versus colorectal), estimation technique (CFA versus BSEM), and questionnaire length (39-item FFMQ versus 20-item FFMQ-SF). Compared with previous validation studies, the present sample of colorectal cancer patients was older, less educated, and had more males. The present study found that patients with more advanced cancer showed higher senses of acting with awareness. The intensity of illness experience and lengthy treatment procedures could induce a heightened sense of mind–body dissonance and higher risks of emotional distress [55] for these patients.

One should note that mindfulness is far from a unitary construct and mindfulness could have both the trait and state components. Dispositional or trait mindfulness is typically assessed by the FFMQ as a self-report questionnaire. Mindfulness practice has been shown to lead to both trait and state changes in mindfulness [56] and disposition towards mindfulness could change over a longer period of time through repeated mindfulness practice. Longitudinal studies with repeated follow-up assessments are needed to distinguish between the intervention effects on dispositional mindfulness and state mindfulness. Future research could explore potential personality, genetic, environmental factors that moderate the effects of mindfulness practice.

### Study limitations

Several limitations of this study should be noted. First, sample sizes equal to four times the number of parameters are needed [44] to achieve accurate parameter estimates and reliable goodness-of-fit tests under Bayesian estimation. The final BSEM model with informative priors suffered from an inadequate N:pD ratio of small sample size (N = 127) to the number of parameters (pD = 109). Despite the proper convergence of all the BSEM models, the low N:pD ratio might still affect the reliability of parameter estimations and goodness-of-fit tests. The use of BSEM with a small sample could inadvertently model idiosyncratic sample characteristics. Both exploratory and confirmatory factor analyses were carried out using data from the same sample. The lack of independent samples suggests the need to confirm the modifications of the factor loadings and residual correlations of the FFMQ-SF in further studies with larger samples.

Second, the convenience sampling method in the present study is subject to common-method and self-selection biases. The lack of meditation experience of the participants limits the generalizability of the results to non-meditators. Further studies are recommended to examine the measurement invariance of the Chinese FFMQ-SF across meditators and non-meditators. Third, the present study did not consider potential cultural factors that influence the role mindfulness plays in the Chinese context [57]. A recent study [58] found more robust relationships between mindfulness and grit in the Western individualistic culture than in the Eastern collectivistic culture. Future research could attempt to elucidate the role of cultural factors from the perspective of Eastern philosophies.

## Conclusion

The present study illustrates the use of BSEM as a flexible analytic approach that incorporates theoretical knowledge via approximate zero informative priors. The present findings only provide partial support for the psychometric properties of the FFMQ-SF in colorectal cancer patients. Overall, the BSEM results supported the five-factor structure with acceptable reliability for four of the five facets and adequate convergent validity for acting with awareness. However, the nonjudging facet showed questionable construct validity, reliability, and convergent validity. Further studies with larger sample sizes are needed to elucidate the viability of FFMQ-SF as a measure of mindfulness facets in cancer patients.

## Supplementary Information

**Additional file 1.** Original 5-factor structure of the FFMQ-SF.

**Additional file 2.** Factor analysis under maximum likelihood estimators.

**Additional file 3.** Supplementary figure on the 5-factor CFA model.

## Data Availability

The dataset analyzed during the current study is available from the corresponding author on a reasonable request.

## Abbreviations

FFMQ: Five Facet Mindfulness Questionnaire
SF: Short-form
BSEM: Bayesian structural equation modeling
PPP: Posterior predictive *p* value
EFA: Exploratory factor analysis
DIC: Deviance information criterion
ICC: Intraclass correlation coefficient
