# Supplementary figures and images for: Psychometric properties of the Chinese version of Five Facet Mindfulness Questionnaire—short form in cancer patients: a Bayesian structural equation modeling approach
Source: Health Qual Life Outcomes. 2021 Feb 10;19:51. doi: 10.1186/s12955-021-01692-1 (PMC7877071; doi:10.1186/s12955-021-01692-1)

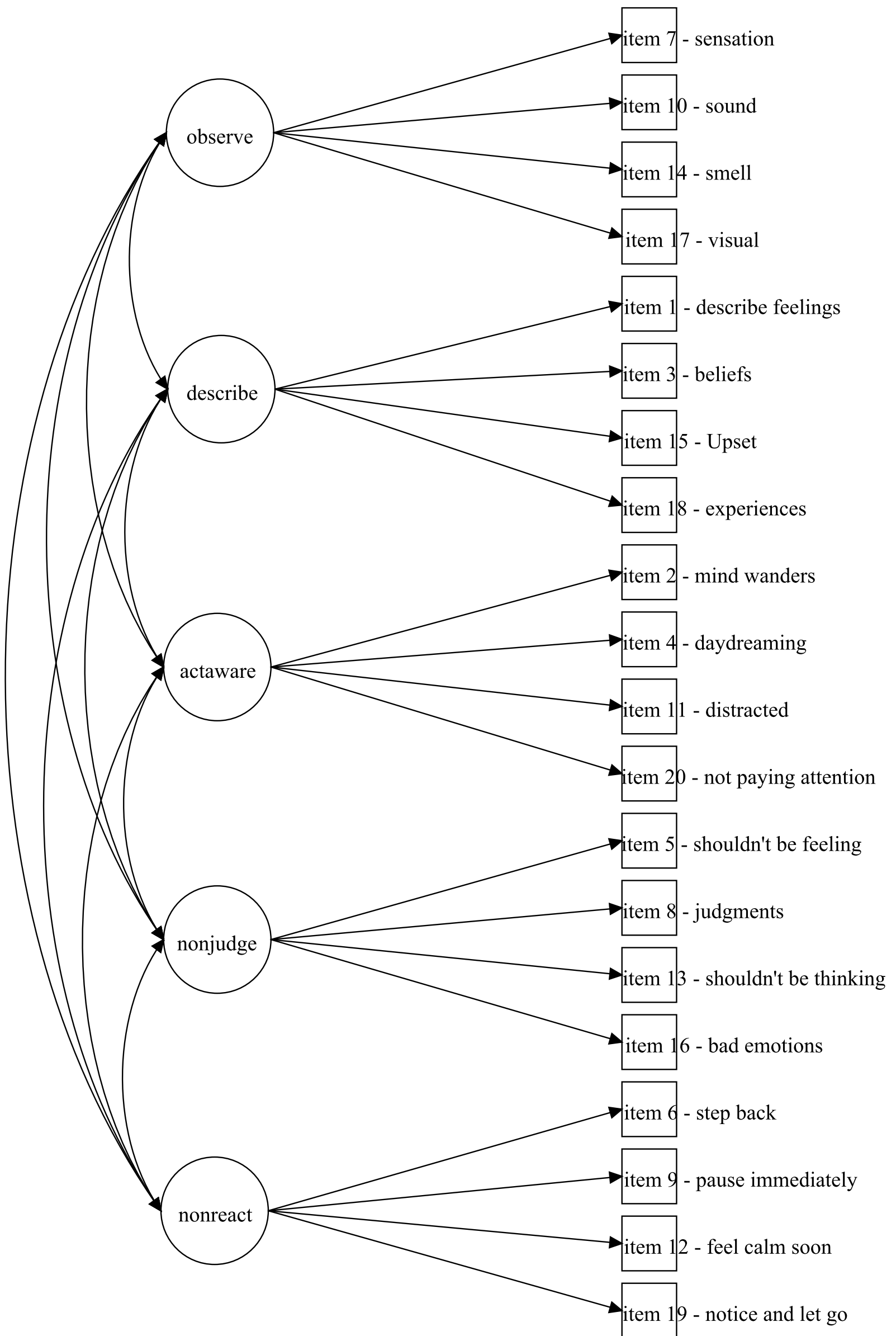

Supplement: Supplementary file 1 — Additional file 1. Original 5-factor structure of the FFMQ-SF. [file 12955_2021_1692_MOESM1_ESM.pdf]

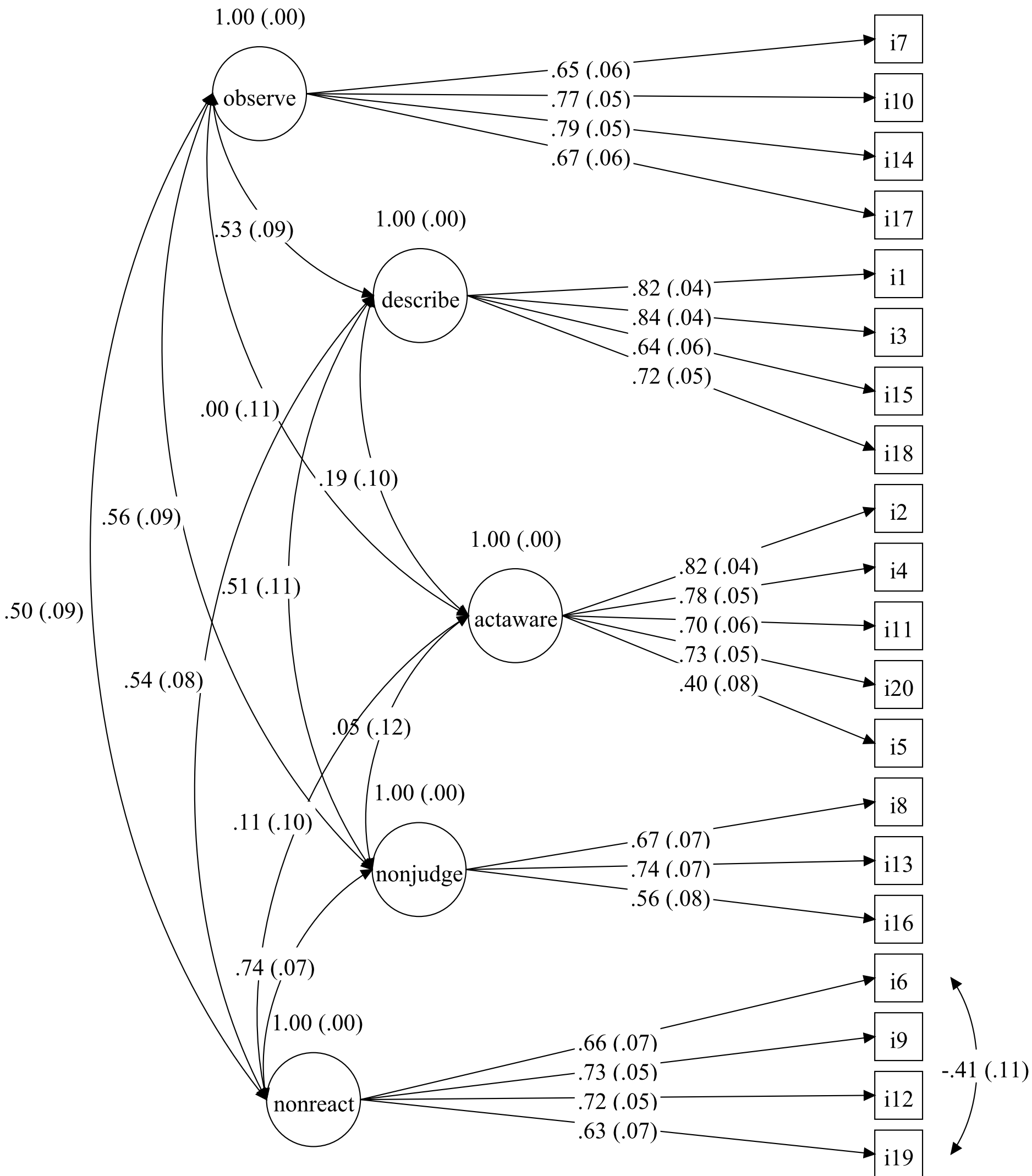

Supplement: Supplementary file 3 — Additional file 3. Supplementary figure on the 5-factor CFA model. [file 12955_2021_1692_MOESM3_ESM.pdf]
