# Supplementary material for: Psychometric properties of the Chinese version of Five Facet Mindfulness Questionnaire—short form in cancer patients: a Bayesian structural equation modeling approach
Source: Health Qual Life Outcomes. 2021 Feb 10;19:51. doi: 10.1186/s12955-021-01692-1 (PMC7877071; doi:10.1186/s12955-021-01692-1)
Supplement: Supplementary file 2 — Additional file 2. Factor analysis under maximum likelihood estimators. [file 12955_2021_1692_MOESM2_ESM.docx]

**Factor analysis under maximum likelihood estimators**

Traditional factor analysis - Methodology

To corroborate the results obtained under the Bayesian approach, we conducted traditional confirmatory factor analysis (CFA) and exploratory factor analysis (EFA) under the maximum likelihood approach. The former should be equivalent to BSEM using uninformative priors and the latter allowed specification of cross-loadings and potential residual covariances. Model fit of the CFA/EFA models was evaluated using the χ^2^ test of exact fit, RMSEA, CFI, and standardized root mean square residuals (SRMR). Cutoff criteria for approximate model fit were RMSEA ≤ 0.06, CFI ≥ .95, and SRMR ≤ 0.06 (Hu and Bentler, 1999). Model comparison was done using Bayesian information criterion (BIC), with a lower BIC value denoting better model fit with greater parsimony.

Traditional factor analysis - Results

As shown in Supplementary Table 1, the traditional 5-factor CFA model provided a poor model fit with χ^2^(160) = 321.6, *p* < 0.001, RMSEA and SRMR > 0.07, and CFI < 0.90. The modified CFA model with added residual covariance between item 6 and item 19 also provided a poor fit to the data. Overall, the CFA models did not account for the potential small cross-loadings and were clearly rejected by the data. Factor loadings of the modified CFA model were provided in the supplementary file.

The EFA model showed considerably higher CFI and lower RMSEA and SRMR than the CFA model. Addition of the item covariance between item 6 and item 19 resulted in an approximate model fit to the data with χ^2^(99) = 146.1, *p* = 0.002, RMSEA ~ 0.06, SRMR < 0.06, and CFI ≥ 0.95. The residual correlation between item 6 and item 19 was significant (*r* = -0.41, p < 0.05).

The two EFA models provided similar fits as Models 5 and 9 under BSEM in Table 4. EFA could be regarded as an exploratory variant of BSEM without a priori factor structure and informative priors. The revised EFA model showed the same factor structure as in the BSEM results.

| **Supplementary Table 1** Fit indices of the 5-factor CFA and EFA models for the FFMQ-SF | | | | | | | | |
| --- | --- | --- | --- | --- | --- | --- | --- | --- |
| Model | # | χ^2^ | *df* | *p* | BIC | RMSEA | CFI | SRMR |
| CFA | 70 | 322 | 160 | .000 | 6764 | .089 | .84 | .090 |
| modified CFA | 71 | 295 | 159 | .000 | 6742 | .082 | .87 | .085 |
|  |  |  |  |  |  |  |  |  |
| EFA | 130 | 154 | 100 | .000 | 6886 | .065 | .95 | .039 |
| EFA + 1 residual cov. | 131 | 146 | 99 | .002 | 6884 | .061 | .95 | .038 |
| N = 127; # = number of free parameters; χ^2^ = chi-square; *p* = p-value for exact fit; BIC = Bayesian information criterion; RMSEA = Root mean square error of approximation; CFI = comparative fit index; SRMR = standardized root mean square residual. | | | | | | | | |
